# Supplementary material for: Correction: The Campylobacter jejuni Cj0268c Protein Is Required for Adhesion and Invasion In Vitro
Source: PLoS One. 2014 Aug 25;9(8):e107076. doi: 10.1371/journal.pone.0107076 (PMC4143353; doi:10.1371/journal.pone.0107076)
Supplement: File S1 — Originally published, uncorrected article. (PDF) [file pone.0107076.s001.pdf]

The *Campylobacter jejuni* Cj0268c Protein Is Required for Adhesion and Invasion *In Vitro*

A. Malik Tareen[1], Carsten G. K. Lüder[1], Andreas E. Zautner[1,2], Uwe Groβ[1], Markus M. Heimesaat[3], Stefan Bereswill[3], Raimond Lugert[1]*

1 University Medical Center Göttingen, Institute for Medical Microbiology, Göttingen, Germany, **2** University Medical Center Göttingen, Department of Clinical Chemistry/Central Laboratory, Göttingen, Germany, **3** Charité – University Medicine Berlin, Department of Microbiology and Hygiene, Berlin, Germany

Abstract

Adherence of *Campylobacter jejuni* to its particular host cells is mediated by several pathogen proteins. We screened a transposon-based mutant library of *C. jejuni* in order to identify clones with an invasion deficient phenotype towards Caco2 cells and detected a mutant with the transposon insertion in gene *cj0268c*. *In vitro* characterization of a generated non-random mutant, the mutant complemented with an intact copy of *cj0268c* and parental strain NCTC 11168 confirmed the relevance of Cj0268c in the invasion process, in particular regarding adherence to host cells. Whereas Cj0268c does not impact autoagglutination or motility of *C. jejuni*, heterologous expression in *E. coli* strain DH5α enhanced the potential of the complemented *E. coli* strain to adhere to Caco2 cells significantly and, thus, indicates that Cj0268c does not need to interact with other *C. jejuni* proteins to develop its adherence-mediating phenotype. Flow cytometric measurements of *E. coli* expressing Cj0268c indicate a localization of the protein in the periplasmic space with no access of its C-terminus to the bacterial surface. Since a respective knockout mutant possesses clearly reduced resistance to Triton X-100 treatment, Cj0268c contributes to the stability of the bacterial cell wall. Finally, we could show that the presence of *cj0268c* seems to be ubiquitous in isolates of *C. jejuni* and does not correlate with specific clonal groups regarding pathogenicity or pathogen metabolism.

Citation: Tareen AM, Lüder CGK, Zautner AE, Groβ U, Heimesaat MM, et al. (2013) The *Campylobacter jejuni* Cj0268c Protein Is Required for Adhesion and Invasion *In Vitro*. PLoS ONE 8(11): e81069. doi:10.1371/journal.pone.0081069

Editor: Dipshikha Chakravortty, Indian Institute of Science, India

Received July 5, 2013; **Accepted** October 9, 2013; **Published** November 26, 2013

Copyright: © 2013 Tareen et al. This is an open-access article distributed under the terms of the Creative Commons Attribution License, which permits unrestricted use, distribution, and reproduction in any medium, provided the original author and source are credited.

Funding: This work was supported by the Deutsche Forschungsgemeinschaft (grant number GR 906/13-1). A. Malik Tareen was funded by a grant from the German Academic Exchange Service (DAAD). Stefan Bereswill and Markus M. Heimesaat are supported by DFG (SFB633, TP A7) (SFB633, TP B6) and by the German Federal Ministery of Education and Research (BMBF) ("Lab in a hanky" projects TP 1.1 and TP 8.2). Furthermore, the authors acknowledge the support by the German Research Foundation and the Open Access Publication Funds of the G?ttingen University. The funders had no role in study design, data collection and analysis, decision to publish, or preparation of the manuscript.

Competing Interests: Markus M. Heimesaat and Stefan Bereswill currently serve as editors for this journal. This does not alter the authors' adherence to all the PLOS ONE policies on sharing data and materials.

* E-mail: rlugert@gwdg.de

Introduction

Campylobacter jejuni is a Gram-negative spiral shaped bacterium, which is pervasive in mammals and birds. The chicken intestine is the natural reservoir that is frequently colonized by the pathogen. In recent years, *C. jejuni* has emerged as an abundant reported cause of bacterial diarrhoea in industrialized as well as developing countries with approximately 2.5 million estimated cases per year in the United States and more than 60,000 annual cases in Germany. Contaminated chicken meat, beef and milk products are common sources of transmission and human infection [1–4]. The progress of the disease can vary from watery to bloody diarrhea including fever and abdominal cramps. In rare cases immunopathological sequelae such as Guillain-Barré syndrome might arise even months or years post infection [5,6].

Adherence of *C. jejuni* to intestinal epithelial cells is essential for successful infection in human hosts. In the past, *in vitro* as well as *in vivo* studies revealed distinct proteins which are important for *C. jejuni* to adhere to its particular target cells. For instance, PEB1, the periplasmic component of an aspartate/glutamate ABC-transporter, mediates adherence and invasion of human epithelial cells and is important for the intestinal colonization of mice [7–9]. Furthermore, the major outer membrane protein MOMP adheres to fibronectin and is involved in binding of *C. jejuni* to the membrane of INT407 cells [10]. CadF, another outer membrane protein with an apparent molecular weight of 37 kDa, and FlpA have also been shown to connect to fibronectin. These interactions, in turn, result in the activation of integrin receptors to launch a host cell signal cascade leading to restructuring of the actin cytoskeleton mediating the uptake of *C. jejuni* [11–15]. The surface-exposed lipoprotein JlpA is required for efficient adherence of the pathogen to HEp-2 epithelial cells and initiates the activation of NF-κB and p38 MAP kinase. Consequently, JlpA seems to be involved in the proinflammatory host cell response upon *C. jejuni* infection [16,17]. Furthermore, CapA, an autotransporter protein of *C. jejuni,* has been shown to be associated with the adherence and invasion of epithelial cells by the pathogen and plays an important role in the colonization of the chicken gut. Recently, it was demonstrated that the bacterial outer membrane protein Cj0091 mediates adherence of *C. jejuni* to INT407 cells and contributes to the colonization of chickens as well [18,19]. Taken together, all these adhesion factors contribute significantly to the interaction between host cell and bacterial pathogen to allow the subsequent process of cellular invasion. In addition to these

proteins described to be involved in the process of adherence, also lipooligosaccharides (LOS) are important for pathogen-host cell interactions given that *C. jejuni* strains deficient in genes involved in LOS metabolism (*wlaRG, wlaTB* and *wlaTC*) exhibited diminished adherence properties to chicken embryo fibroblasts [20]. Recently, our group generated a *C. jejuni* mutant deficient in sulphite:cytochrome c oxidoreductase (SOR) which exhibited a down-regulated transcription of genes involved legionaminic acid synthesis and possessed reduced adherence properties to Caco2 cells [21]. Finally, a recently identified *C. jejuni* type VI secretion system (T6SS) was shown to be involved in cell adhesion. Following functional knockout of the T6SS-genes *hcp1* and *icmF1*, the adherence capacity of the respective mutants to T84 colon epithelial cells was reduced by approximately 50% as compared to the parental strain [22].

In this report we describe the *C. jejuni* gene *cj0268c* which has been shown by us and others to be important for the capability of the pathogen to infect host cells [23,24]. This protein with a molecular weight of 40.2 kDa and an isoelectric point of 8.93 possesses a putative transmembrane domain around amino acid 60 and a SPFH domain encompassing the amino acids 64 to 259. Proteins containing the stomatin/prohibitin/flotillin/HflK/C (SPFH) domain can be found in divergent species ranging from bacteria to mammals. The precise function of this domain, however, is still unclear even though mammal proteins containing SPFH domains are frequently found in lipid raft microdomains within several cellular membranes [25–27]. In support of this, Hinderhofer *et al.* [28] could categorize altogether 1090 SPFH domain-containing proteins from 497 different bacterial species encompassing all phyla into 12 subfamilies. However, despite the knowledge acquisition about the evolutionary development of prokaryotic SPFH proteins, the general biological function of this motif is still unclear. Here we demonstrate that Cj0268c is required for adhesion of *C. jejuni* to different host cells. Heterologous expression revealed its potential to alter the adhesion capacity of *E. coli*. The exact subcellular localization of Cj0268c was not known yet. Although analysis of the protein sequence using SignalIP 4.1 (Technical University of Denmark) revealed no evidence for the presence of a signal peptide, flow cytometry analysis after immunolabeling of Cj0268c in *E. coli* indicated the protein to reside in the periplasmic space with no exposure of the C-terminus at the bacterial surface. Furthermore, we determine the relevance of Cj0268c regarding motility, autoagglutination, the resistance of *C. jejuni* to bile salts and the stability of the bacterial cell to the nonionic surfactant Triton X-100. Finally, we investigate the affiliation of *cj0268c* to particular clonal groups of *C. jejuni*.

Materials and Methods

Bacterial strains, media and culture conditions

C. jejuni strain B2 initially isolated from a patient suffering from gastroenteritis [29,30] and strain NCTC 11168 were grown on Columbia agar supplemented with 5% defibrinated sheep blood at 42°C in microaero- and capnophilic conditions (85% N_2, 10% CO_2, 5% O_2). If required, appropriate antibiotic concentrations of kanamycin (50 µg ml^{-1}) or chloramphenicol (30 µg ml^{-1}) were added. Growth experiments were carried out at 42°C in Mueller-Hinton (MH) broth under microaero- and capnophilic conditions. *Escherichia coli* strain DH5α was grown on Luria bertani (LB) agar or broth at 37°C. When necessary, ampicillin (100 µg ml^{-1}) was added. Growth experiments were carried out at 42°C in Mueller-Hinton (MH) broth under the above mentioned conditions for 24 h.

Generation of competent cells and electroporation

10 ml of LB-broth were inoculated with a single *E. coli* DH5α colony and incubated overnight at 37°C under shaking. Three ml of the overnight culture were grown in 100 ml LB-broth at 37°C to an $OD_{(600\ nm)}$ of 0.35–0.45. The culture was transferred into a 50 ml Falcon tube, placed on ice for 10 min and centrifuged for 15 min at 4000×g at 4°C. Then the cell pellet was gently resuspended in 30 ml ice cold TFB1 buffer and incubated on ice for 30 min. Cells were pelleted by centrifugation as described above. Then, the cell pellet was carefully dissolved in 2 ml ice-cold TFB2 buffer and incubated on ice for another 30 min. After incubation aliquots of 100 µl were stored at −80°C.

C. jejuni, cells were harvested from Columbia blood agar plates and centrifuged at 5,000×g at 4°C for 10 minutes. After washing of the *C. jejuni* cells three times in 1 ml ice-cold wash buffer containing 272 mM sucrose and 15% glycerol at 4°C, the pellet was resuspended in 400 µl washing buffer and 100 µl aliquots were used for electroporation, respectively.

For each transformation 0.5 to 3 µg of plasmid DNA were added to an aliquot of competent cells in an ice-cold electroporation cuvette. After incubation of the cuvette containing the mixture of bacteria and DNA, electroporation was performed at 2.5 kV, 25 µF and 200 Ω using the BTX Electro Cell Manipulator. While adding 500 µl of SOC medium to transform *E. coli*, in case of *C. jejuni*, the suspension was transferred onto a non selective Columbia blood agar plate and incubated overnight at 37°C under microaerophilic conditions. Finally, cells were transferred onto a selective plate and incubated at 42°C under microaerophilic conditions for additional 2–3 days. *E. coli* were plated directly on selective LB agar containing the appropriate antibiotic agent and incubated at 37°C overnight.

Cultivation of cells

Human colon carcinoma Caco2 cells were cultivated in Dulbecco minimal essential medium (DMEM) supplemented with 10% fetal bovine serum (FBS), 1 x non-essential amino acids, 100 U ml^{-1} penicillin, and 100 µg ml^{-1} streptomycin. Primary chicken cecal (PCC) cells, kindly provided by Ingrid Hänel, were isolated as described elsewhere [31] and maintained in Quantum 286 medium for epithelial cells (PAA Laboratories) supplemented with 5% chicken serum and 0.5% chick embryo extract. Both cell lines were incubated in a humidified atmosphere of 95% air and 5% CO_2 at 37°C.

Isolation of nucleic acids

Genomic DNA of *C. jejuni* was isolated with the QIAamp DNA Mini Kit (Qiagen) according to the instructions of the manufacturer. Plasmid DNA was prepared using the GeneElute Plasmid Miniprep Kit (Sigma) following the manufacturer's protocol.

Insertional knockout of gene *cj0268c*

Initially, using genomic DNA from strain NCTC 11168 as a template, a 1090 bp DNA fragment representing *cj0268c* was amplified with primers Cj0268cF and Cj0268cR (Table 1). The obtained PCR product was *XbaI* digested and ligated to the *XbaI* restricted and dephosphorylated plasmid vector pBluescript II KS (Stratagene). For the subsequent knockout, inverse PCR with the primers Cj0268cinvF and Cj0268cinvR using the plasmid described above as a template was carried out. The PCR reaction containing 10 mM Tris-HCl pH 8.3, 50 mM KCl, 1.5 mM $MgCl_2$, all four dNTPs (each 0.2 mM) and 10 pmol of each primer was carried out using 1 U PfuUltra High-Fidelity DNA Polymerase (Stratagene) to obtain a blunt end PCR product.

Initial incubation at 95°C for 1 min was followed by initial 10 cycles at 95°C for 30 s, 55°C for 30 s and 72°C for 5 min, followed by 25 cycles under the same conditions with the exception of a shifted annealing temperature to 58°C. The resulting PCR product with a length of 3752 bp comprised of the complete cloning vector pBluescript, the 413 5′-terminal nucleotides and the 378 3′-terminal nucleotides of *cj0268c*. After gel extraction of the PCR product using the QIAquick PCR Purification Kit (Qiagen), a phosphorylated blunt end kanamycin resistance cassette described previously [21] was ligated with the obtained PCR product using Quick Ligase (New England Biolabs) following the instructions of the manufacturer to obtain plasmid pBcj0268c-kanR.

Cloning of gene *cj0268c* into pRRC

For functional complementation of the *C. jejuni cj0268c*-knockout strain, gene *cj0268c* which was PCR-amplified with primers Cj0268cF and Cj0268cR and *XbaI* restricted as described above was cloned into likewise digested and dephosphorylated *C. jejuni* expression vector pRRC [32] to obtain pRRC-*cj0268c*. In order to provide *cj0268c* with a His-tag, the gene was amplified with primers Cj0268cF and Cj0268cHis, which are listed in Table 1. PCR was performed in a TRIO-Thermocycler (Biometra) with 10 ng of genomic DNA of *C. jejuni* as a template. The PCR mixture contained 1 U PfuUltra High-Fidelity DNA Polymerase (Stratagene), 1x inherent reaction buffer, dNTPs (each 0.2 mM) and 10 pmol of each primer. After initial incubation at 95°C for 3 min, 40 cycles at 95°C for 30 s, 55°C for 30 s and 72°C for 2 min were carried out with a final incubation at 72°C for 5 min. Afterwards, the PCR amplicon was *XbaI*-digested and cloned into pRRC as mentioned above.

Adhesion and invasion assays

Bacterial invasion assays were performed according to the publication of Everest *et al.* [33]. In brief, Caco2 cells were grown to approximately 80% confluence in a 6 well plate, washed with PBS and inoculated with 400 µl *C. jejuni* suspension adjusted to an $OD_{(600\ nm)}$ of 0.5 which corresponds to a multiplicity of infection (MOI) of 100. To investigate invasion, the *C. jejuni* suspension was removed after two hours and the cells were washed three times with PBS before further incubation with culture medium supplemented with 100 µg ml^{-1} gentamicin. For the release of intracellular bacteria, the cells were lysed with 1% Triton X-100 for 10 min and the number of viable bacteria was determined by counting the number of bacteria colony forming units (cfu) grown on Columbia blood agar plates after incubation for 48 h at 42°C under microaerophilic conditions. For investigating bacterial adhesion, 6 well plates containing Caco2 cells and inoculated with *C. jejuni* or *E. coli* bacteria were centrifuged at 600×g for 5 min to increase the association of the bacteria with the cells. After incubation for 30 min, the monolayers were washed with PBS, cells were lysed and subsequently, plating of the bacteria was performed as described above. Every experiment was repeated four times.

Motility and autoagglutination assays

Tests for altered motility or autoagglutination of the *C. jejuni* strains and mutants were carried out as described previously by Tareen *et al.*, 2011 [21] and Tareen and Dasti *et al.*, 2010 [24].

Resistance of *C. jejuni* to bile salts

Bacteria were harvested from overnight-incubated blood agar plates and adjusted to an $OD_{600\ nm}$ of 0.1 in Muller Hinton broth. Then, the bacterial cultures were supplemented with 0.09, 0.18, 0.37, 0.75, 1.5, and 3% of cholate and deoxycholate, respectively. After incubation for 24 h at 37°C in a microaerophilic atmosphere, $OD_{600\ nm}$ was determined. All tests were carried out in triplicate.

Sensitivity of *C. jejuni* to Triton X-100

The sensitivity of *C. jejuni* to Triton X-100 was analyzed using bacterial colonies grown overnight which were harvested from

Table 1. Oligonucleotide primers used for sequencing, cloning of the *C. jejuni* gene *cj0268c* with and without His-tag, generation of the knockout mutant and screening of genomic DNA samples for the presence of *cj0268c*.

Gene	Primer name	Sequence (5′ to 3′)
sequencing		
aphA-3	KanF	TATCACCTCAAATGGTTCGCT
cj0268c	Cj0268cseq	CAGCGCCAAAGGTAAAGC
cloning		
cj0268c	Cj0268cF	GCTCTAGAAAAAGGAAATAA**ATG**CCAGCTGATTTG
	Cj0268cR	GCTCTAGA**TTA**GTTCATGTTGGCAGCACTTTGCTTT
	Cj0268cHis	GCTCTAGA**TTA***GTGATGGTGATGGTGATG*GTTCATGTTGGCAGCACTTTGCTTT
	Cj0268cinvF	ACAGGCAATCCAGCAGAGTC
	Cj0268cinvR	AGAACAAATCGAACGCGTGC
kanR	Kan1	**P**-GTAAGATTATACCGAGGTATGAAAACG
	Kan2	**P**-AATCTAGGTACTAAAACAATTCATCCA
screening		
cj0268c	268cGMF	GTACAGCGCGAAGTTCTACA
	268cGMR	TCGTGATGTAGTGCGAAGTG

XbaI restriction sites for cloning in pRRC and pBluescript II KS are underlined, the start and the stop codons of *cj0268c* are shown in bold and the nucleotide sequence corresponding to the His-tag is illustrated in *italic*. Primers Kan1 and Kan2 are 5′-phosphorylated. The *Cj* number refers to the homolog in the genome of *C. jejuni* strain NCTC11168.
doi:10.1371/journal.pone.0081069.t001

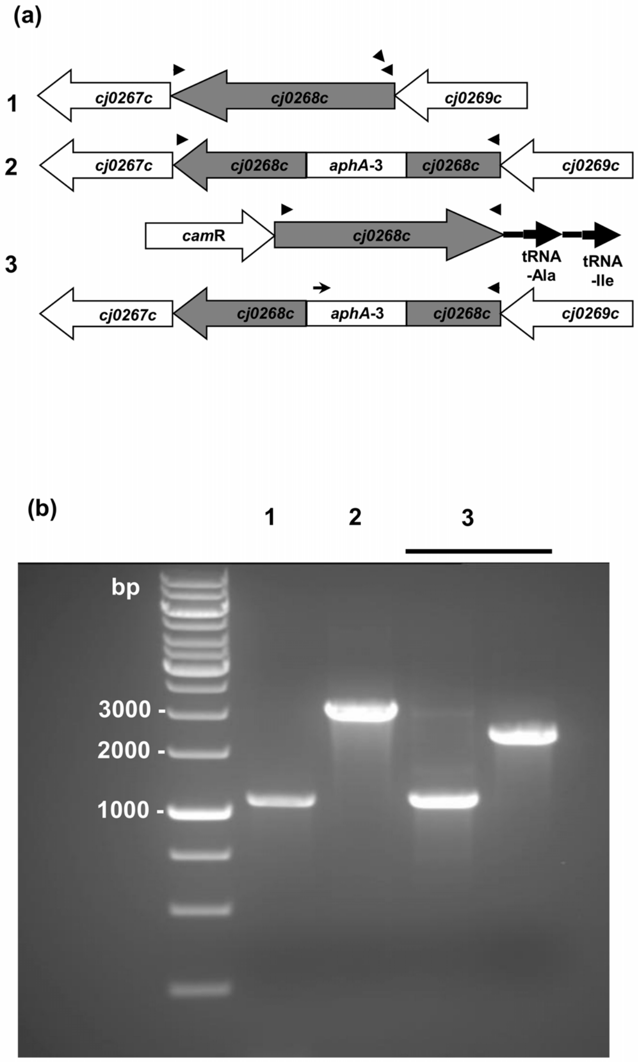

Figure 1. Generation of a *cj0268c*-knockout mutant and a complemented mutant in *C. jejuni* strain NCTC 11168. (1) parental strain NCTC 11168, (2) *cj0268c* knockout mutant (NCTC 11168::*cj0268c*), (3) complemented knockout mutant (NCTC 11168::*cj0268c*-comp-*cj0268c*). (a) Genome arrangements of the bacterial strains under investigation. Primers Cj0268cF and Cj0268cR for the amplification of*cj0268c* are indicated by arrowheads. The primer KanF that binds to the 5′-end of the kanamycin resistance cassette is shown by an arrow. (b) Verification of the native gene, the respective mutant and the complemented mutant strain by PCR. (1) PCR analysis with Cj0268cF + Cj0268cR-primers detect the native gene of 1090 bp. (2) *aphA-3* insertion in gene *cj0268c* mediating kanamycin resistance in mutant strain NCTC 11168::*cj0268c* was verified by the amplification of a 2914 bp PCR- amplicon applying primers Cj0268cF+R. (3) the complemented mutant was verified by PCR analysis with primers Cj0268cF+R to detect the native gene (1090 bp) and with primers KanF + Cj0268cR which amplify a PCR product of 2168 bp.
doi:10.1371/journal.pone.0081069.g001

blood agar plates. The bacteria were resuspended in distilled water and adjusted to an $OD_{600\ nm}$ of 0.1. Triton X-100 was added to a final concentration of 1.0%, and after incubation for one hour at room temperature the number of viable bacteria was determined by plating serial dilutions onto blood agar plates and subsequent incubation at 42°C for 48 h in a microaerophilic atmosphere. Every experiment was repeated three times.

Screening of *C. jejuni* strains for presence of gene *cj0268c*

Genomic DNA samples of 56 *C. jejuni* strains were analyzed by PCR with 10 pmol of primers 268cGMF and 268cGMR (Table 1) and 1 U *Taq*-Polymerase (Roche) in a TRIO-Thermocycler (Biometra) under following conditions. After an initial denaturation at 95°C for 1 min, 35 cycles at 95°C for 30 s, 55°C for 30 s and 72°C for 1 min were performed. Afterwards, samples were run on a 2% agarose gel for the detection of the resulting 146 bp PCR amplicon.

Lysis of *E. coli* DH5α cells and immunoblot analysis

E. coli from 5 ml overnight culture were centrifugated and resuspended in 500 µl 1x PBS. After addition of 100 µg lysozyme and NP40 to a final concentration of 1%, lysates were incubated on ice and were casually shaken for 10 min. Following the addition of 22 µl of 5 M NaCl, the lysates were centrifuged for 45 min at 16000×g. Then, the supernatants were subject of subsequent immunoblot assays. Aliquots of 20 µl were separated in a 15% SDS-PAGE gel and blotted onto a PVDF membrane (GE Healthcare) by a semidry transport system (Sartorius). After protein transfer, the membrane was blocked for 1 h with 5% milk powder in PBS containing 0.05% Tween 20. Subsequent incubation of the membrane with a 1:3000 dilution of monoclonal mouse anti-His primary antibody (Qiagen) over night at 4°C was followed by labelling of the immune complexes with a 1:3000 dilution of horseradish-peroxidase-conjugated anti mouse secondary antibody (Dianova) for 1 h at room temperature and visualization by ECL chemiluminescence.

Permeabilization of *E. coli* DH5α cells

In order to get access to the bacterial periplasm, *E. coli* cells were permeabilized. prior to antibody labelling and flow cytometry analysis. 5×10^7 bacterial cells of each sample were washed in 1 x PBS and were resuspended in 180 µl 20% sucrose, 50 mM Tris-HCl, pH 8 containing 10 mM EDTA. After 10 min 20 µl lysozyme at a final concentration of 2 mg/ml in 20 mM Tris-HCl, pH 8, 2 mM EDTA, 1.2% NP40 were added, and the samples were incubated at 37°C for 20 min. Finally, cells were washed twice in 20% sucrose, 50 mM Tris-HCl, pH 8.

Antibody labelling of *E. coli* cells and flow cytometric measurements

Unspecific antibody binding was prevented by incubation of *E. coli* cells in 100 µl 1 x PBS containing 1% BSA for 30 min at 4°C. Cells were collected by centrifugation at 10.000 g for 2 min and incubated in 100 µl 1 x PBS, 1% BSA containing anti-penta His antibody (Qiagen) at a concentration of 10 µg/ml at 4°C for 30 min. After washing three times with 1 x PBS, 1% BSA, immune complexes were labelled with 50 µl R-phycoerythrin-conjugated goat $F(ab')_2$ fragment anti-mouse IgG (1:50, Dianova) for 30 min at 4°C. After having been washed, cells were fixed in 1% PFA in PBS. Subsequently, flow cytometric measurements were carried out using a FACSCalibur flow cytometer (Becton Dickinson).

Statistical analysis

The significance of differences (*P*-value less than 0.01) between mean values was calculated by the Mann-Whitney U test.

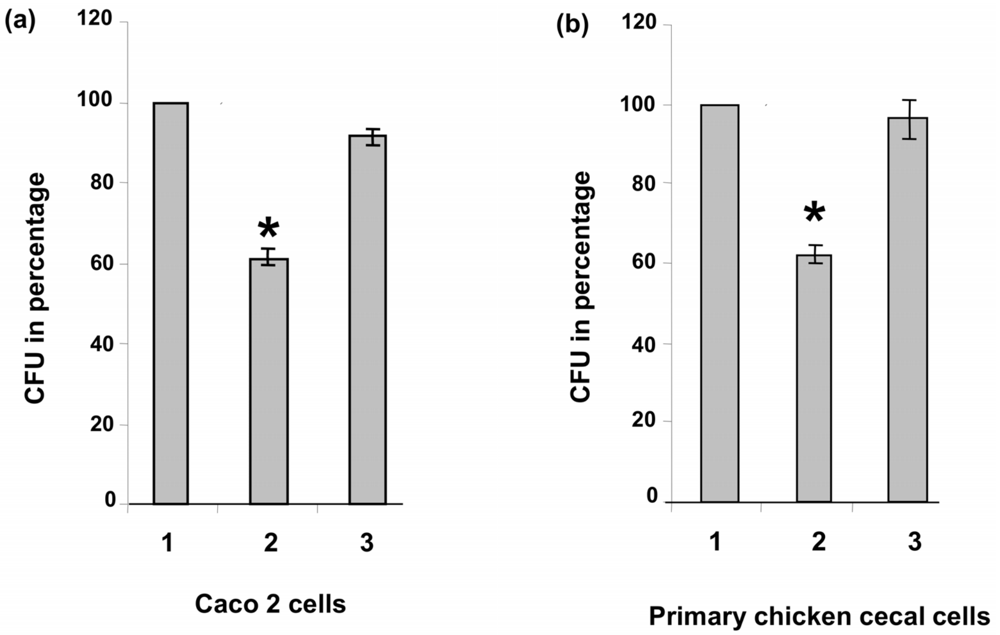

Figure 2. Gentamicin protection assays. 1. wild type strain NCTC 11168, 2. mutant strain NCTC 11168::*cj0268c*, 3. complemented mutant NCTC 11168::*cj0268c*-comp-*cj0268c*. Gentamicin protection assays with the strains under investigation using (a) Caco2 cells and (b) PCC-cells. The assays on Caco2 cells with the parental strain, the knockout mutant and the complemented knockout mutant confirmed the infection-deficient phenotype to be due to the functional loss of *cj0268c*. Four independent experiments have been carried out, respectively. The standard deviations are indicated. (a) Taking the number of *C. jejuni* wild type strain NCTC 11168 colonies recovered as 100%, the mean value of *cj0268c*-mutant colonies (NCTC 11168::*cj0268c*) accounted for 62% (±2.41), whereas the percentage of obtained colonies from the complemented strain (NCTC 11168::*cj0268c*-comp-*cj0268c)* was 93% (±2.04). Since the *P*-value for the mutant was less than 0.001, the reduced invasion capacity was significant. (b) The relevance of *cj0268c* for invasion is not restricted to human cells, since it could also be shown for the invasion of PCC-cells. Compared to the invasion capacity of parental strain NCTC 11168 (100%), the percentage of colonies obtained from mutant strain NCTC 11168::*cj0268c was* only 62% (±2.36, $P<0.0007$). However, complementation of the mutant strain with *cj0268c* restored the infectivity of NCTC 11168::*cj0268c*-comp-*cj0268c* which was similar to that of the parental strain (96%, ±4.96).
doi:10.1371/journal.pone.0081069.g002

Results

Knockout of *cj0268c* in *C. jejuni* reference strain NCTC 11168 and functional complementation

In a previous report we detected gene *cj0268c* to contribute to the invasion of host cells in *C. jejuni* strain B2 [24]. Since strain B2 is highly invasive in human colon epithelial cells but fails to infect chicken cells or to colonize birds we wanted to confirm the invasion deficient phenotype in the *C. jejuni* reference strain NCTC 11168 [34]. This strain is capable of causing gastroenteritis in men as well as settling in livestock and, for this, enables us to test whether *cj0268c* has a defined function for colonization or infection of a particular host. After introduction of plasmid pBcj0268c-kanR to obtain NCTC 11168::*cj0268c* we restored the parental phenotype by transformation of the mutant strain with pRRC-*cj0268c* to obtain NCTC 11168::*cj0268c*-comp-*cj0268c*. A description of the genetic arrangement of *cj0268c* in strain NCTC 11168, the *cj0268c* knockout mutant, the complemented strain and the location of the primers is shown in Figure 1a.

In order to independently confirm the invasion deficient phenotype of *C. jejuni* strain B2Δ*cj0268c* in reference strain NCTC 11168, we repeated the gentamicin protection assays on Caco2 cells with the wild type strain, the *cj0268c*-knockout mutant and the corresponding complemented strain. Thereby, the phenotype of strain B2Δ*cj0268c* could be approved and, hence, the invasion-deficient phenotype demonstrated to be due to the functional loss of *cj0268c* in both, the *C. jejuni* strains B2 as well as in NCTC 11168 [24]. If the number of recovered NCTC 11168 wild type colonies is defined as 100%, we detected a mean value of *cj0268c*-mutant colonies of only 62% ($p<0.0007$). The percentage of obtained colonies from the complemented strain was 93% which was not significantly different from values of the parental NCTC 11168 strain (Fig. 2a). To address, whether the invasion-deficient phenotype due to the loss of *cj0268c* was restricted to human cells, we performed gentamicin protection assays with primary chicken cecal cells infected with strains NCTC11168, NCTC11168::*cj0268c*, and NCTC 11168::*cj0268c*-comp-*cj0268c*. In support of our data obtained with Caco2 cells, the invasion capacity of the *cj0268c*-mutant strain was significantly reduced compared to parental NCTC 11168 strain, but was completely restored in the complemented mutant. If the invasion capacity of wild type strain NCTC 11168 was defined as 100%, the percentage of colonies obtained from mutant strain NCTC 11168::*cj0268c was* only 62% (±2.36, $P<0.0007$) but was reconstituted to 96% (±4.96) by the intact gene in the complemented strain NCTC 11168::*cj0268c*-comp-*cj0268c* compared to NCTC 11168 (Fig. 2b).

Adherence

Given that *cj0268c* encodes a putative transmembrane protein, we next tested whether this protein is involved in the pathogen-host cell adherence process. Applying adhesion assays with wild type strain NCTC 11168, the *cj0268c*-mutant and the complemented mutant on Caco2 cells, we could decisively detect an adherence-deficient phenotype of NCTC 11168::*cj0268c* compared to NCTC 11168 which could be restored to wild type level in the *cj0268c*-complemented strain. When defining the number of

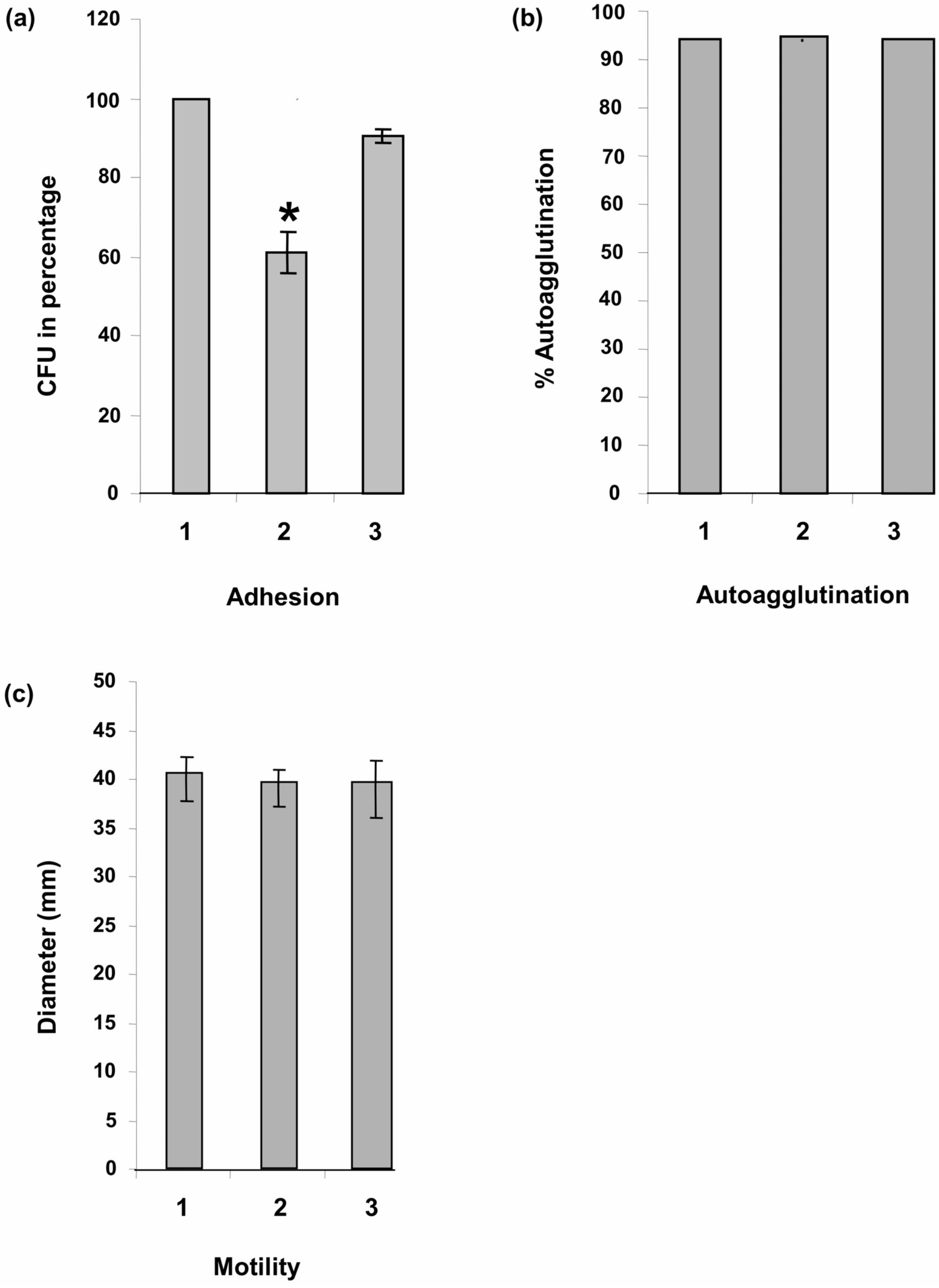

Figure 3. Assays to test for adhesion, autoagglutination and motility of the strains under investigation. (1) parental strain NCTC 11168, (2) *cj0268c* knockout mutant (NCTC 11168::*cj0268c*), (3) complemented knockout mutant (NCTC 11168::*cj0268c*-comp-*cj0268c*). (a) Loss of *cj0268c* reduces the capability of *C. jejuni* to adhere to Caco2 cells. Defining the number of wild type strain NCTC 11168 colonies obtained performing adhesion assays as 100%, the mean value of colonies from the mutant NCTC 11168::*cj0268c* was 60.9% (±5.18, P<0.0039) in contrast to NCTC 11168::*cj0268c*-comp-*cj0268c* with a recovery rate of 96.4% (±1.77). (b, c) Gene *cj0268c* does neither impair the property of *C. jejuni* to autoagglutinate nor the motility of the pathogen. By performing corresponding tests, no differences among the bacterial strains under investigation could be detected. Both, the percentage of autoagglutinated bacteria and the motility zones did not show any significant differences between wild type, mutant and complemented mutant.
doi:10.1371/journal.pone.0081069.g003

colonies recovered from the parental strain NCTC 11168 as 100%, the mean value of corresponding colonies for the mutant NCTC 11168::*cj0268c* was 60.9% (±5.18, P<0.0039) whereas the recovery rate of colonies for the complemented strain was 96.4% (±1.77) which represents the wild type adhesion level (Fig. 3a).

To further phenotypically characterize the *cj0268c*-deficient mutant, we performed assays to test for altered motility or an affected capacity to autoagglutinate compared to the wild type strain and the complemented mutant. As shown in Figures 3b and 3c the mutant strain exhibited the same motility and autoagglu-

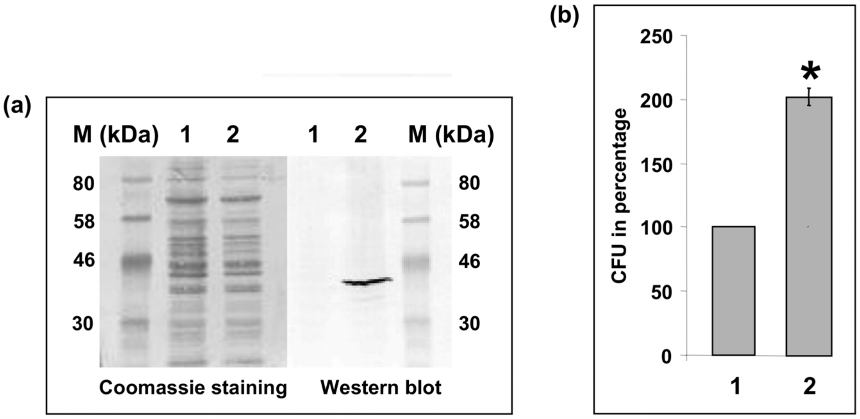

Figure 4. Detection of Cj0268c in *E. coli* and altered adherence. 1. *E. coli* DH5α/pRRC, 2. *E. coli* DH5α/pRRC-cj0268cHis. (a) Immunoblot with a monoclonal antibody against the His-tag detected a protein with a molecular weight of 41 kDa corresponding to Cj0268c. The protein was exclusively found in the lysate of the Cj0268c-expressing *E. coli*. The respective Coomassie staining served as a loading control. (b) Adherence assays on Caco2 cells with Cj0268c-expressing *E. coli* and *E. coli* transformed with plasmid pRRC alone. Taken the number of recovered *E. coli* colonies from the strain harbouring pRRC without *cj0268c* as 100%, the mean value of the adherence capacity of *E. coli* DH5α/pRRC-cj0268cHis was 201.7% (±6.44, P<0.0039).
doi:10.1371/journal.pone.0081069.g004

tination properties as compared to NCTC 11168 wildtype or NCTC 11168::*cj0268c*-comp-*cj0268c* and, thereby, excluding a role of *cj0268c* regarding these characteristics.

E. coli strain DH5α expressing Cj0268c possesses an increased adherence to Caco2 cells

After cloning of *cj0268c* including a C-terminal His-tag in pRRC, we were able to detect recombinant Cj0268c expression also in *E. coli* DH5α even though in this vector the chloramphenicol resistance-mediating gene, as well as *cj0268c* is under control of the *C. jejuni* 16S promoter. Performing Western-Blot analysis with corresponding *E. coli* DH5α lysates and a monoclonal antibody against the His-tag, we determined a specific protein band of an approximate size of 41 kDa which represents the expected size of Cj0268c (Fig. 4a). To investigate if Cj0268c expressed in the heterologous context of *E. coli* intensifies its capability to interact with eukaryotic cells, we repeated the adherence assays with Caco2 cells. By defining the number of recovered *E. coli* DH5α colonies representing the parental strain as 100%, the relative recovery rate of live bacterial colonies from the Cj0268c-expressing *E. coli* was 201.7% (±6.44). Hence, we could confirm the adherence-mediating phenotype of Cj0268c, indicating that Cj0268c does not necessarily need to interact with other

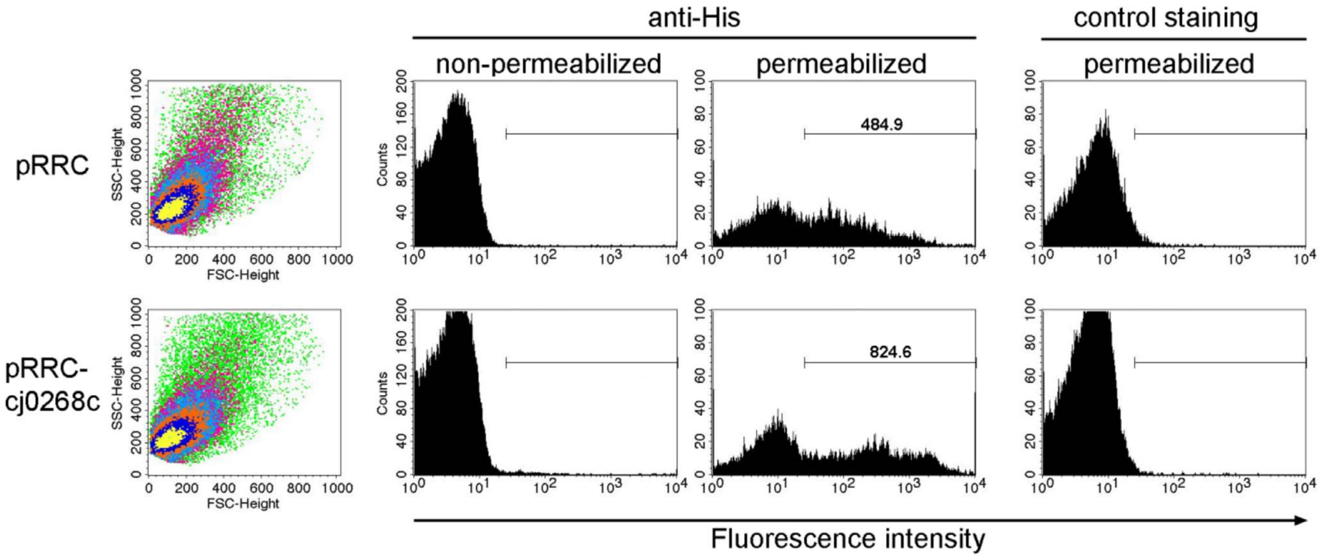

Figure 5. Flow cytometric measurements of permeabilized and non-permeabilized *E. coli* cells expressing Cj0268c (pRRC-*cj0268c*) or transformed with an empty vector (pRRC). Bacteria were immunolabelled with a monoclonal anti-pentaHis primary antibody and a R-phycoerythrin-conjugated secondary antibody. E. coli cells of both populations were gated according to the forward scatter and the side scatter (dot plots, left panel). Permeabilized *E. coli* cells harbouring the empty plasmid pRRC without gene *cj0268c* (upper panel) possess a considerable lower fluorescence intensity as compared to an *E. coli* population which express Cj0268c (lower panel). To verify the specificity of binding of anti-pentaHis, a control staining with the secondary antibody only was included (right panel).
doi:10.1371/journal.pone.0081069.g005

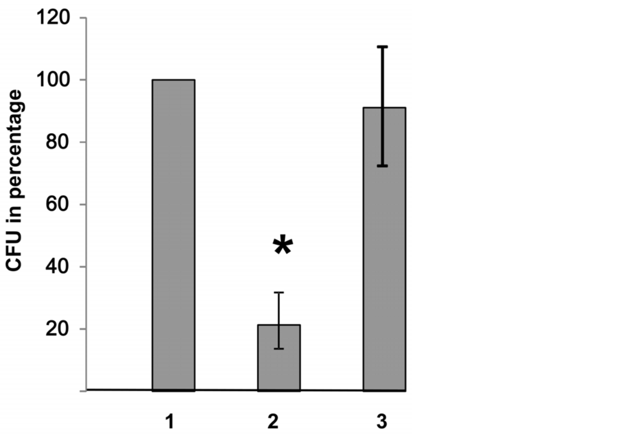

Figure 6. Resistance to Triton X-100. (1) Parental strain NCTC 11168, (2) *cj0268c* knockout mutant (NCTC 11168::*cj0268c*), (3) complemented knockout mutant (NCTC 11168::*cj0268c*-comp-*cj0268c*). The numbers of colonies of the respective fourth dilution plated onto blood agar are shown. The *cj0268c* knockout mutant shows a clearly diminished resistance to Triton X-100. When defining the number of wild type colonies as 100%, the mean value of colonies recovered from the corresponding *cj0268c*-mutant was 21.3% (±9.02, P<0.0039). After complementation of the mutant with an intact copy of *cj0268c* the percentage of colonies obtained increased to 91.3%.
doi:10.1371/journal.pone.0081069.g006

C. jejuni proteins, but by itself enhances adhesion properties in a heterologous host (Fig. 4b).

Localization of Cj0268c

In order to determine whether Cj0268c is localized at the bacterial surface we carried out flow cytometric measurements with non-permeabilized *E. coli* cells. After incubation of *E. coli* expressing a His-tagged version of Cj0268c with a monoclonal anti-His primary antibody followed by labelling with R-Phycoerythrin-conjugated secondary antibody, we could not detect any particular staining of the bacterial cell surface compared to *E. coli* bacteria that harboured plasmid pRRC without gene *cj0268c* (Fig. 5). Hence, at least the His-tagged C-terminus of Cj0268c is not localized at the bacterial surface. In contrast, when we permeabilized the bacterial cell wall with EDTA and lysozyme to allow access of the antibodies to the proteins of the periplasm, we could clearly detect immunolabelling of the *E. coli* population expressing Cj0268c. Whereas anti-His labelling was also obtained after labelling of permeabilized *E. coli* which had been transformed with the empty vector, the mean fluorescence intensity of antibody-labelled cells was considerably higher for Cj0268c-expressing cells as compared to controls (824.6 FLI units as compared to 484.9; Fig. 5). Control staining with the secondary antibody only of both Cj0268c-positive and negative *E. coli* confirmed the specificity of the FACS analysis. Together, these results indicate that the protein Cj0268c resides in the periplasmic space.

Resistance to bile salts and Triton X-100

Next we determined whether the presence of Cj0268c strengthens the resistance against bile salts and detergents. Strains NCTC11168, NCTC11168::*cj0268c*, and NCTC 11168::*cj0268c*-comp-*cj0268c* were incubated with different concentrations of cholate and deoxycholate ranging from 0.09 to 3%. Subsequent measurements of the optical density of the cultures revealed no differences between wildtype, *cj0268c*-mutant and its complemented mutant. Given that we were not able to detect any bacterial growth in the presence of bile salt concentrations exceeding 0.75%, further experiments were carried out only with cholate and deoxycholate concentrations up to 0.75%. However, the measurements of the optical densities in these experiments did not reveal any significant differences neither comparing the respective bacterial strains nor with respect to different bile salt concentrations tested (data not shown). Thus, these data suggest that Cj0268c does not exert a functional correlation with e.g. efflux pump systems such as CmeABC, for instance.

To find out whether Cj0268c has any influence on the stability of the bacterial cell wall, we incubated the *cj0268c*-deficient strain, its complemented version and NCTC 11168 wildtype strain in the presence of the nonionic surfactant Triton X-100 which is commonly used as a detergent to permeabilize cellular membranes. Thereby, the *cj0268c* mutant strain was much more sensitive to Triton X-100 as compared to wild type and complemented mutant. When we incubated the respective strains in a final concentration of 1% Triton X-100 for 1 h, subsequent plating onto Columbia blood agar yielded significantly fewer CFU of the *cj0268c*-mutant strain as compared to wild type strain NCTC 11168 and the *cj0268c*-complemented version. When defining the number of wild type colonies obtained after plating of the fourth dilution as 100%, the relative abundance of *cj0268c*-mutant CFU yielded only a mean of 21.3% (Fig. 6), indicating that Cj0268c significantly contributes to the bacterial cell stability.

The *cj0268c* gene is ubiquitous in the *C. jejuni* population

In recent studies multilocus sequence typing (MLST) analysis of defined genetic markers allowed the classification of different *C. jejuni* clonal groups. In addition, the combination of these genetic markers correlated, at least to some degree, with a corresponding animal source [35]. Moreover, after expansion of the MLST analysis by the inclusion of further gene markers, an association with a higher prevalence of campylobacterioses in humans or livestock adaption could be assigned regarding to the distribution of these markers [36]. We therefore studied next whether gene *cj0268c* fits into one of these clonal groups and, furthermore, could be associated with the *C. jejuni* settlement of particular animal groups or the severity of human campylobacteriosis. Altogether 56 isolates out of the 266 isolates used in the studies mentioned above from all clonal groups were screened for the presence of gene *cj0268c* by PCR. We were able to detect gene *cj0268c* in all isolates of *C. jejuni* indicating that *cj0268c* is not related to a distinct clonal group but rather seems to be ubiquitous in *C. jejuni* (not shown). Thus, a contribution of *cj0268c* to the *C. jejuni* settlement of specific animal groups or the clinical course of campylobacteriosis is unlikely.

Discussion

Screening of our transposon-generated mutant library of *C. jejuni* strain B2 revealed altogether seven genes that mediate a diminished invasion capacity towards Caco2 cells as shown by gentamicin protection assays [24]. Gene *cj0268c* which was further characterized here has been shown to be involved in host cell invasion earlier [23]. In order to investigate this gene regarding its biological function in further detail, we inactivated *cj0268c* in *C. jejuni* reference strain NCTC 11168. This strain, in contrast to strain B2, is able to infect both, human as well as chicken cells. After generation of a corresponding *cj0268c*-complemented NCTC 11168 strain, we could verify this gene to belong to a number of factors which are important for the adhesion to host cells by the pathogen. Furthermore, we confirmed this role of

Cj0268c by heterologous expression of *cj0268c* in *E. coli* strain DH5α. This result confirmed that Cj0268c possesses an adhesion mediating function alone and does not have to interact with other proteins of *C. jejuni*. On the other hand, an alteration of surface properties of *E. coli* after heterologous expression of Cj0268c leading to an indirect effect cannot be ruled out. Since we obtained equal CFUs of the *E.coli* populations independent of the expression of Cj0268c after incubation for 10 min in the presence of 1% Triton X-100, we could exclude alterations in bacterial stability caused by Cj0268c as the reason for different CFU numbers. However, since the adherence of *C. jejuni* to human and chicken cells depended exclusively on the presence or absence of Cj0268c irrespective of the specific host cell species, we determined Cj0268c as a protein for the mediation of adherence in general.

The chicken intestine represents a natural habitat for *C. jejuni* colonization and, hence, resistance to bile salts is essential for the pathogen's survival in such a hostile milieu. One of the resistance mechanisms exerted by *C. jejuni* employs the multidrug efflux pump system CmeABC, consisting of an outer membrane protein CmeC, a drug transporter CmeB, localized in the inner membrane, and periplasmic CmeB to connect CmaA and CmeC [37–39]. Since Cj0268c is a predicted transmembrane protein, interaction with CmeB to stabilize the CmeABC complex for instance was conceivable. However, after incubation of parental strain NCTC 11168 and its corresponding *cj0268*-mutant, the resistance to bile salts like cholate and deoxycholate exerted by the pathogen strains was virtually identical. Triton X-100 incubation of *C. jejuni* strains with mutated genes leading to an incomplete lipooligosaccharide metabolism had an inconsistent outcome reported so far. Whereas a mutation in the heptosyltransferase gene *waaF (cj1148)* decreased the stability of the *C. jejuni* cell wall, the resistance to Triton X-100 of the galactosysltransferase *cj1136*-deficient mutant was not altered compared to the parental strain [40,41]. Nevertheless in the case of Cj0268c, incubation of the parental strain, the mutant and the complemented mutant with 1% Triton X-100 revealed that Cj0268c is important for maintaining integrity of the bacterial cell wall. Finally, although we could clearly demonstrate Cj0268c to possess an adherence-mediating function and, therefore, contributes to the invasion process, the corresponding gene seems to be ubiquitous and not to belong to distinct clonal *C. jejuni* groups which could be related to pathogenicity according to earlier findings of [34,35].

The *in vitro* results presented here need to be further complemented by investigating the biological impact of the respective mutant strains in a suitable murine model mimicking human campylobacteriosis.

Acknowledgments

We thank Ingrid Hänel (FLI, Jena, Germany) for providing PCC cells, and we thank the CASVAB, University of Balochistan, Pakistan and the Higher Education Commission, Pakistan.

Author Contributions

Conceived and designed the experiments: RL AMT UG. Performed the experiments: AMT AEZ CGKL. Analyzed the data: AMT RL MMH SB CGKL. Contributed reagents/materials/analysis tools: AMT RL AEZ UG. Wrote the paper: RL UG MMH SB.

References

1. Altekruse SF, Stern NJ, Fields PI, Swerdlow DL (1999) *Campylobacter jejuni*-an emerging foodborne pathogen. Emerg Infect Dis 5: 28–35.
2. Mead GC, Slutsker L, Dietz V, McCaig LF, Breese JS, et al. (1999) Food-related illness and death in the United States. Emerg Infect Dis 5: 607–625.
3. Friedman CR, Neiman J, Wegener HC, Tauxe RV (2000) Epidemiology of *Campylobacter jejuni* in the United States and other industrialized nations. In *Campylobacter*, 2nd edn Edited by I. Nachamkin & M. J. Blaser. Washington DC: American Society for Microbiology. pp. 121–138.
4. Dasti JI, Tareen AM, Lugert R, Zautner AE, Groβ U (2010) *Campylobacter jejuni*: a brief overview on pathogenicity-associated factors and disease-mediating mechanisms. Int. J. Med. Microbiol 300: 205–211.
5. Allos BM (2001) *Campylobacter jejuni* infections: update on emerging issues and trends. Clin Infect Dis 32: 1201–1206.
6. Schmidt-Ott R, Schmidt H, Feldmann S, Brass F, Krone B, et al. (2006) Improved serological diagnosis stresses the major role of *Campylobacter jejuni* in triggering Guillain-Barré syndrome. Clin Vaccine Immunol 13: 779–783.
7. Pei ZM, Blaser J (1993) PEB1, the major cell-binding factor of *Campylobacter jejuni* is a homolog of the binding component in gram-negative nutrient transport systems. J Biol Chem 268: 18717–18725.
8. Pei Z, Burucoa C, Grignon B, Bagar S, Huang XZ, et al. (1998) Mutation in the peb1A locus of *Campylobacter jejuni* reduces interactions with epithelial cells and intestinal colonization of mice. Infect Immun 66: 938–943.
9. Leon-Kempis Mdel R, Guccione E, Mulholland F, Williamson MP, Kelly DJ (2006) The *Campylobacter jejuni* PEB1a adhesin is an aspartate/glutamate-binding protein of an ABC transporter essential for microaerobic growth on dicarboxylic amino acids. Mol Microbiol 60: 1262–1275.
10. Moser I, Schroeder W, Salnikow J (1997) *Campylobacter jejuni* major outer membrane protein and a 59-kDa protein are involved in binding to fibronectin and INT 407 cell membranes. *FEMS* Microbiol Lett 157: 233–238.
11. Konkel ME, Garvis SG, Tipton SL, Anderson DE Jr, Cieplak W Jr (1997) Identification and molecular cloning of a gene encoding a fibronectin-binding protein (CadF) from *Campylobacter jejuni*. Mol Microbiol 24: 953–963.
12. Krause-Gruszczynska M, Rohde M, Hartig R, Genth H, Schmidt G, et al. (2007) Role of the small Rho GTPases Rac1 and Cdc42 in host cell invasion of *Campylobacter jejuni*. Cell Microbiol 9: 2431–2444.
13. Flanagan RC, Neal-McKinney JM, Dhillon AS, Miller WG, Konkel ME (2009) Examination of *Campylobacter jejuni* putative adhesins leads to the identification of a new protein, designated FlpA, required for chicken colonization. *Infect Immun* 77: 2399–2407.
14. Konkel ME, Larson CL, Flanagan RC (2010) *Campylobacter jejuni* FlpA binds fibronectin and is required for maximal host cell adherence. J Bacteriol 192: 68–76.
15. Eucker TP, Konkel ME (2012) The cooperative action of bacterial fibronectin-binding proteins and secreted proteins promote maximal *Campylobacter jejuni* invasion of host cells by stimulating membrane ruffling. Cell Microbiol 14: 226–238.
16. Jin S, Joe A, Lynett J, Hani EK, Sherman P, et al. (2001) JlpA, a novel surface-exposed lipoprotein specific to *Campylobacter jejuni*, mediates adherence to host epithelial cells. Mol Mirobiol 39: 1225–1236.
17. Jin S, Song YC, Emili A, Sherman PM, Chan VL (2003) JlpA of *Campylobacter jejuni* interacts with surface-exposed heat shock protein 90alpha and triggers signalling pathways leading to the activation of NF-kappaB and p38 MAP kinase in epithelial cells. Cell Microbiol 5: 165–174.
18. Ashgar SS, Oldfield NJ, Wooldridge KG, Jones MA, Irving GJ, et al. (2007) CapA, an autotransporter protein of *Campylobacter jejuni*, mediates association with human epithelial cells and colonization of the chicken gut. J Bacteriol 189: 1856–1865.
19. Oakland M, Jeon B, Shen Z, Zhang Q (2011) Functional characterization of a lipoprotein-encoding operon in *Campylobacter jejuni*. PLoS ONE 6(5): e20084. doi:10.1371/journal.pone.0020084
20. Holden KM, Gilbert M, Coloe PJ, Li J, Fry BN (2012) The role of WlaRG, WlaTB and WlaTC in lipooligosaccharide synthesis by *Campylobacter jejuni* strain 81116. Microb Pathog 52: 344–252.
21. Tareen AM, Dasti JI, Zautner AE, Groβ U, Lugert R (2011) Sulfite: cytochrom c oxidoreductase deficiency in *Campylobacter jejuni* reduces motility, host cell adherence and invasion. Microbiology 157: 1776–1785.
22. Lertpiriyapong K, Gamazon ER, Feng Y, Park DS, Pang J, et al. (2012) *Campylobacter jejuni* type VI secretion system: roles in adaption to deoxycholic acid, host cell adherence, invasion, and in vivo colonization. PLoS ONE 7(8): e42842. doi:10.1371/journal.pone.0042842
23. Novik V, Hofreuther D, Galán JE (2010) Identification of *Campylobacter jejuni* genes involved in its interaction with epithelial cells. Infect Immun 78: 3540–3553.
24. Tareen AM, Dasti JI, Zautner AE, Groβ U, Lugert R (2010) *Campylobacter jejuni* proteins Cj0952c and Cj0951c affect the chemotactical behavior towards formic acid and are important for the invasion of host cells. Microbiology 156: 3123–3135.10.
25. Morrow IC, Parton RG (2005) Flotillins and the PHB domain protein family: rafts, worms and anaesthetics. Traffic 6: 725–740.
26. Rivera-Milla E, Stuermer CA, Málago-Trillo E (2006) Ancient origin of reggie (flotillin), reggie-like, and other lipid-raft proteins: convergent evolution of the SPFH domain. Cell Mol Life Sci 63: 343–357.
27. Browman DT, Hoegg MB, Robbins SM (2007) The SPFH domain-containing proteins: more than lipid raft markers. Trends Cell Biol 17: 394–402.

28. Hinderhofer M, Walker CA, Friemel A, Stuermer CAO, Möller HM, et al. (2009) Evolution of prokaryotic SPFH proteins. BMC Evol Biol 9: 10.
29. Schmidt-Ott R, Pohl S, Burghard S, Weig M, Groβ U (2005) Identification and characterization of a major subgroup of conjugative *Campylobacter jejuni* plasmids. J Infect 50: 12–21.
30. Dasti JI, Groβ U, Pohl S, Lugert R, Weig M, et al. (2007) Role of plasmid-encoded tet(O) gene in tetracycline-resistant clinical isolates of *Campylobacter jejuni* and *Campylobacter coli*. J Med Microbiol 56: 833–837.
31. Boehm M, Haenel I, Hoy B, Brøndsted L, Smith TG, et al. (2013) Extracellular secretion of protease HtrA from *Campylobacter jejuni* is highly efficient and independent of its protease activity and flagellum. EU-JMI 3: 163–173.
32. Karlyshev AV, Wren BM (2005) Development and application of an insertional system for gene delivery and expression in *Campylobacter jejuni*. Appl Environ Microbiol 71: 4004–4013.
33. Everest PG, Goossens H, Butzler JP, Lloyd D, Knutton S, et al. (1992) Differentiated Caco-2 cells as a model for enteric invasion by *Campylobacter jejuni* and *C. coli*. J Med Microbiol. 37: 319–329.
34. Parkhill J, Wren B, Mungall K, Ketley J, Churcher C, et al. (2000) The genome sequence of the food-borne pathogen *Campylobacter jejuni* reveals hypervariable sequences. Nature 403: 665–668.
35. Zautner AE, Herrmann S, Corso J, Tareen AM, Alter T, et al. (2011) Epidemiological association of different *Campylobacter jejuni* groups with metabolism-associated genetic markers. Appl Environ Microbiol 77: 2359–2365.
36. Zautner AE, Ohk C, Tareen AM, Lugert R, Groβ U (2012) Epidemiological association of different *Campylobacter jejuni* groups with pathogenicity-associated genetic markers. BMC Microbiol 12: 171.
37. Lin J, Overbye ML, Zhang Q (2002) CmeABC functions as a multidrug efflux system in *Campylobacter jejuni*. Antimicrob Agents Chemother 46: 2124–2131.
38. Pumbwe L, Piddock LJ (2002) Identification and molecular characterization of CmeB, a *Campylobacter jejuni* multidrug efflux pump. FEMS Microbiol Lett 206: 185–189.
39. Lin J, Martinez A (2006) Effect of efflux pump inhibitors on bile resistance and *in vivo* colonization of *Campylobacter jejuni*. J Antimicrob Chemother 58: 966–972.
40. Kanipes MI, Holder LC, Corcoran AT, Moran AP, Guerry P (2004) A deep-rough mutant of *Campylobacter jejuni* 81-176 is non-invasive for intestinal epithelial cells. Infect Immun 72: 2452–2455.
41. Javed MA, Cawthraw SA, Baig A, Li J, McNally A, et al. (2012) Cj1136 is required for lipooligosaccharide biosynthesis, hyperinvasion, and chick colonization by *Campylobacter jejuni*. Infect Immun 80: 2361–2370.
